# Evaluation and management of a spontaneous corneal rupture secondary to pellucid marginal degeneration, using swept-source anterior segment optical coherence tomography

**DOI:** 10.1093/omcr/omab003

**Published:** 2021-03-08

**Authors:** Esther Papamlichael, Abison Logeswaran, Vasilios P Papastefanou, Martin Watson, Andrew Coombes

**Affiliations:** 1 Department of Ophthalmology, Bart’s Health NHS Trusts, Royal London Hospital, London, UK; 2 Moorfields Eye Hospital NHS Foundation Trust, London, UK

## Abstract

We describe a case of bilateral spontaneous corneal perforation secondary to pellucid marginal degeneration and present the associated swept-source anterior segment optical coherence tomography (SS-ASOCT) findings and management principles used.

A 47-year-old woman presented with ocular pain, redness, foreign body sensation and clear discharge in the right eye in 2017 and with very similar symptoms in 2019 in the left eye. Clinically she had a corneal perforation at the inferior cornea with associated loss of anterior chamber volume. Corneal topography demonstrated peripheral thinning and steepening in the contralateral eye. ASOCT images revealed full-thickness perforation, iridocorneal touch and iris stranding. The patient was managed with a combination of contact bandaging and corneal gluing.

SS-ASOCT is a useful adjunctive tool in the clinical assessment and evaluation of spontaneous corneal perforation. Alongside the clinical evaluation, it can be used to monitor the clinical response.

## INTRODUCTION

We present a case in which swept-source anterior segment ocular coherence tomography (SS-ASOCT) was used to contribute to the diagnosis and aid in determining the therapeutic management of a patient with spontaneous corneal perforation secondary to pellucid marginal degeneration (PMD). To the best of our knowledge, the incidence of perforation in PMD is not reported in the literature, but one large case series of 116 eyes has reported an incidence of hydrops of 6% [1].

## CASE REPORT

The patient, a 47-year-old Somali woman, presented with a 3-day history of right-sided sharp ocular pain, clear discharge, redness and foreign body sensation. There was no history of trauma, allergy, contact lens use or potential exposure to infection. There was no past medical or ocular history of note.

**Figure 1:**
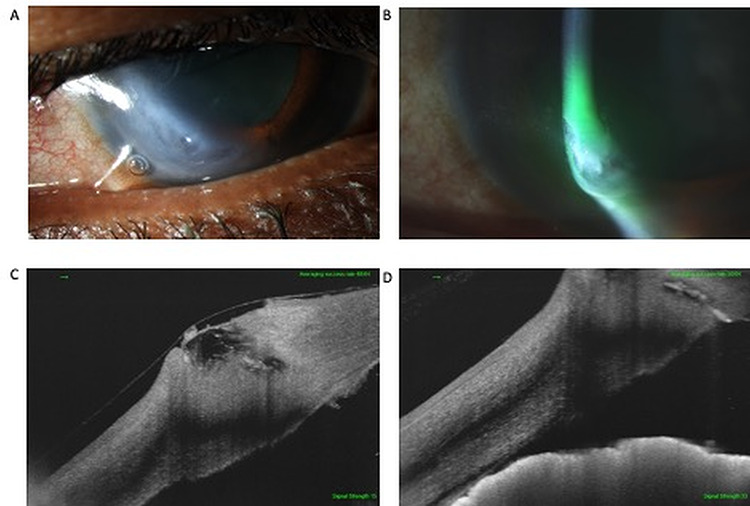
Inferiotemporal peripheral cornea with a bandage contact lens *in situ* (**A**) and associated localized edema (**B**). SS-ASOCT image revealed the presence of an oblique corneal perforation and localized corneal edema (**C**, **D**) as well as iridocorneal touch near the site of perforation (D).

On examination, pinhole visual acuity was 0.6LogMAR and 0.1LogMAR in the right and left eye, respectively. Assessment of the right eye showed an inferior rupture of Descemet’s membrane and a perforation at the 7 o’clock position. There was associated iridocorneal touch, shallow anterior chamber and iris stranding to the site of rupture. On administration of 2% fluorescein there was a positive Seidel’s test that highlighted low flow leakage of aqueous (Fig. 1A and B). Examination of the contralateral eye revealed evidence of inferior corneal thinning.

The patient was investigated with SS-ASOCT (DRI OCT Triton, Japan) that confirmed the presence of a corneal perforation, iridocorneal touch and an iris strand extending to the perforation (Fig. 1C and D).

**Figure 2:**
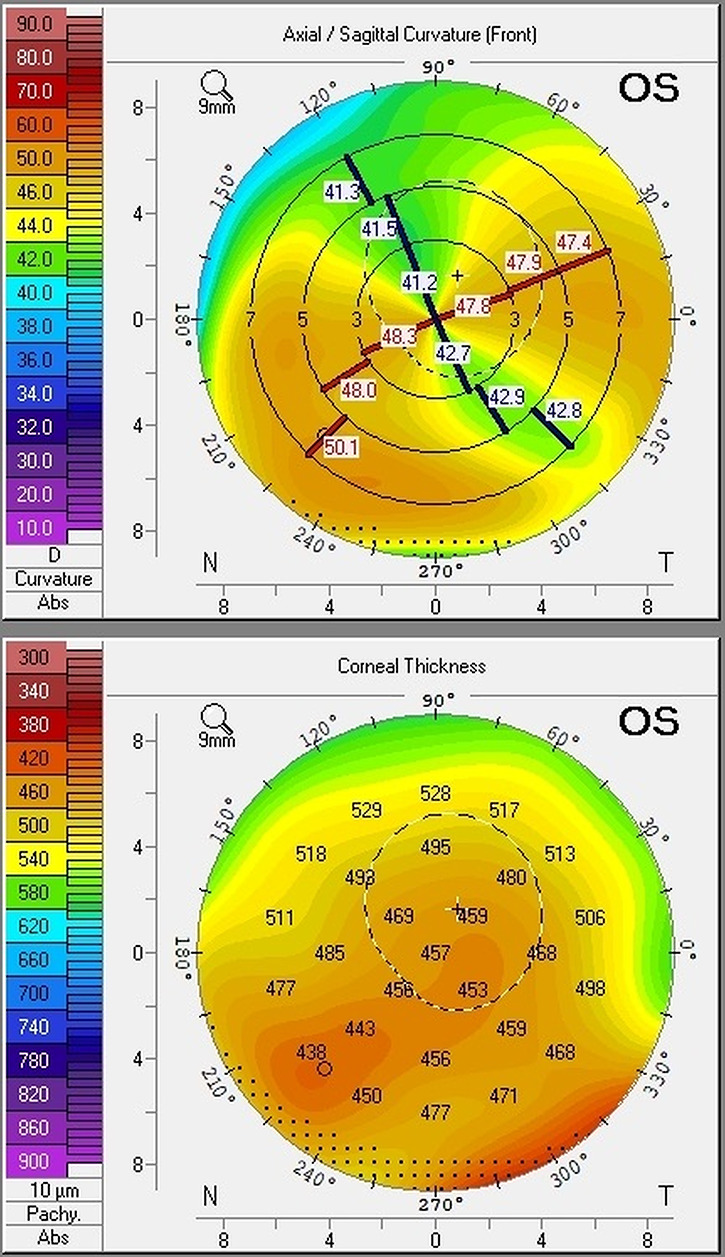
Pentacam^®^ (Oculus) corneal topography of the contralateral eye demonstrating evidence of peripheral steepening and corneal thinning consistent with a diagnosis of PMD.

Pentacam^®^ (Oculus) topography of the contralateral cornea showed evidence of peripheral steepening and thinning consistent with a diagnosis of PMD (Fig. 2). Blood was obtained to screen for potential systemic disorders associated with corneal perforation (full blood count, urea and electrolytes, erythrocyte sedimentation rate, C-reactive protein, full vasculitic and syphilis screen). In the absence of systemic features, normal blood tests and contralateral thinning an underlying systemic disease was felt to be unlikely.

**Figure 3:**
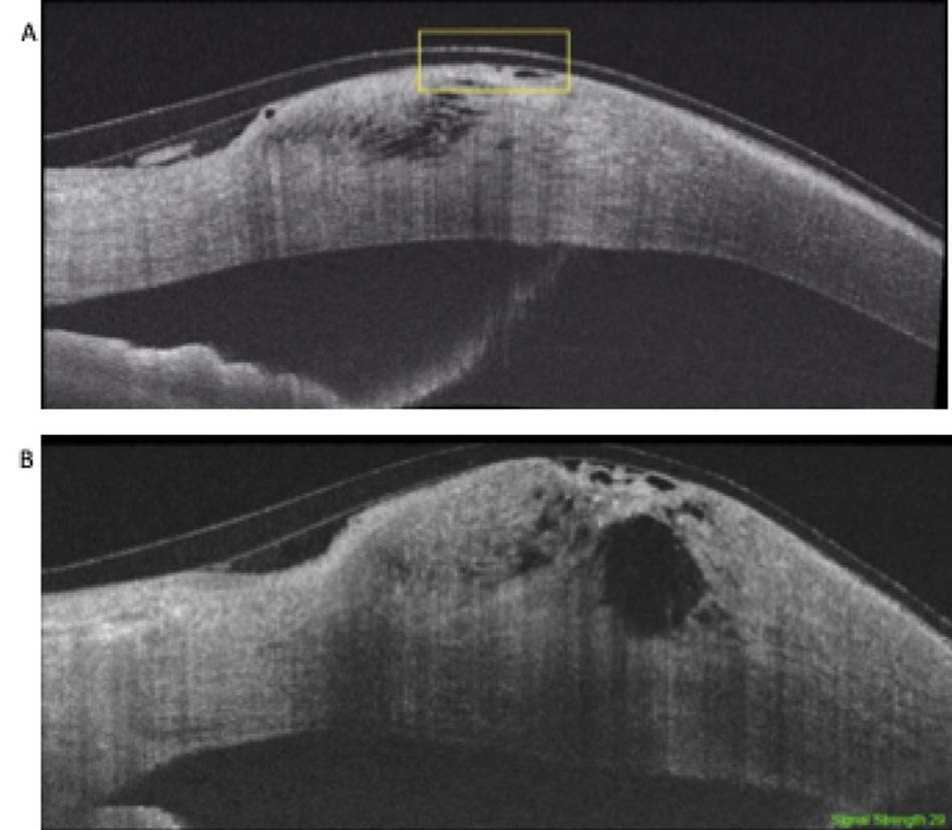
Seven-week follow-up SS-ASOCT images showing partial resolution of the corneal perforation in the posterior stroma with remaining disorganization anteriorly. Peripheral thinning is also demonstrated in these images consistent with the diagnosis of PMD.

In view of a relatively well-formed anterior chamber, absent lenticular corneal touch and low flow leakage, the leak was managed with a bandage contact lens was (PureVision2™, Bausch & Lomb, USA). In addition, she was given oral ciprofloxacin, topical levofloxacin and atropine. A review 24 h later showed a more formed anterior chamber, no iridocorneal touch and reduced rate of aqueous leakage. Topical steroids were added to her current regime and she was reviewed after 72 h.

At this time point the patient had a relatively well-formed anterior chamber, but there was ongoing leakage from the site of perforation. Due to the risk of infection, the patient had corneal cyanoacrylate glue applied with subsequent bandaging.

**Figure 4:**
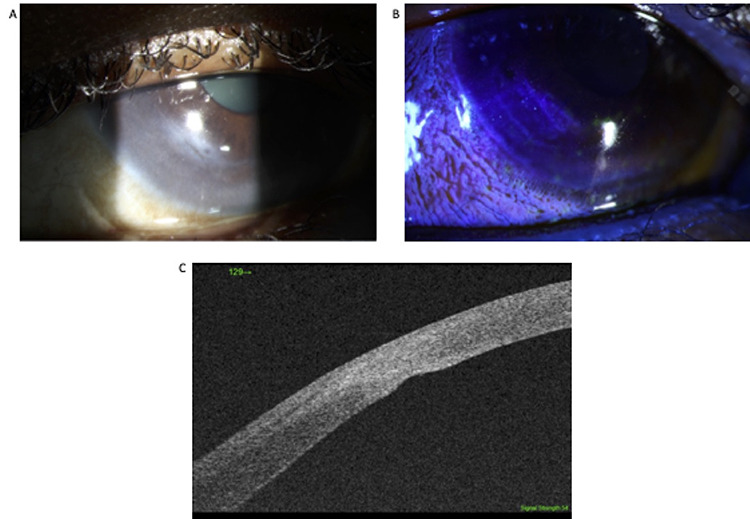
Nine-week anterior segment follow-up pictures showing that the perforation healed (**A**) with no leakage and no residual epithelial deficit (**B**). This was confirmed with SS-ASCOT showing the formation of stromal scar in the area previously perforated and localized thinning (**C**).

She was reviewed on a weekly basis for 7 weeks and during this period required two further gluing procedures. Corneal status was monitored with SS-ASOCT (Fig. 3A and B). At 9 weeks after initial presentation the perforation healed with no leakage and a small residual epithelial deficit (Fig. 4A and B). This was confirmed with SS-ASCOT (Fig. 4c).

The patient presented again 2 years after the original perforation with very similar symptoms in her fellow eye. Examination revealed an inferior rupture of Descemet’s membrane and a perforation at the 7 to 8 o’clock position with a brisk leak. ASOCT confirmed the perforation, and this was managed in the first instance with corneal gluing and anterior chamber reformation in theaters. This failed; so the patient subsequently had primary suturing of the perforation. At 12 weeks postoperatively her corneal sutures were removed uneventfully. Her visual acuity improved to 0.3LogMAR on pin hole in both eyes with a mild cataract.

## DISCUSSION

The underlying causes of corneal perforations are varied but can be broadly characterized into infectious and noninfectious causes. The noninfectious causes are related to four major pathologies: (i) severe ocular surface disease, (ii) autoimmune disease, (iii) trauma and (iv) corneal ectasias.

The clinical and topographical evidence of peripheral corneal thinning suggest that this is the most likely underlying mechanism of perforation in this case. To the best of the authors’ knowledge this is the first reported case of spontaneous corneal perforation secondary to PMD that has been visualized using SS-ASCOT. It provided high-resolution imaging to assess the extent of perforation, degree of wound healing and eventual resolution.

The use of SS-OCT is widespread in the medical retina community due to its improved resolution (1 μm) and ability to image the choroid compared to spectral-domain OCT. The Triton SS-ASOCT 16-mm radius protocol provides a quick scan of the entire cornea, whereas the 6-mm scan provides a more detailed image of a selected area. This is useful for evaluating structural alterations that may not be detectable with larger scans. A number of studies have evaluated ASOCT features of acute hydrops with common findings of epithelial and stromal edema, intrastromal fluid and Descemet’s membrane detachment [2]. Recently SS-ASOCT was used to evaluate corneas with LASIK flap-related complications [3] and as adjunctive tool in evaluating the adherence of grafts post DMEK [4]. It has also been shown to be an effective screening tool for the detection of early subclinical keratoconus [5].

While there is a general consensus for the management of larger perforations (>3 mm), the management of smaller perforations is not well defined. However, there are some important underlying principles. All patients should be advised to avoid touching the eye and an eye shield should be applied. Fluoroquinolones have been shown to have high transcorneal penetration, low rates of resistance and reduced hospital stays [6]. Cycloplegic agents can also be used in conjunction because they provide comfort and minimize adhesions [7]. The use of steroids is controversial. It has the potential to exacerbate infection but could minimize damage from the inflammatory response. Cohen [8] support the notion that steroids should be started after the antibiotic has had time to take effect and the sensitivities are known. The use of a bandage contacts lenses has been shown to promote epithelial healing and minimize discomfort. Cyanoacrylate glue has been shown to improve visual outcomes and prevent progression to perforation in thinned noninfective corneas [9].
